# Microbial vectoring capacity by internal‐ and external‐infesting stored product insects after varying dispersal periods between novel food patches: An underestimated risk

**DOI:** 10.1002/ece3.11368

**Published:** 2024-06-25

**Authors:** Marco A. Ponce, Jacqueline M. Maille, Ian Stoll, Avery James, Alexander Bruce, Tania N. Kim, Erin D. Scully, William R. Morrison

**Affiliations:** ^1^ Department of Entomology Kansas State University Manhattan Kansas USA; ^2^ Department of Biomedical Sciences University of Missouri Columbia Missouri USA; ^3^ Division of Biology Kansas State University Manhattan Kansas USA; ^4^ Department of Plant Pathology and Entomology University of Tennessee Knoxville Tennessee USA; ^5^ USDA, Agricultural Research Service, Center for Grain and Animal Health Research Manhattan Kansas USA

**Keywords:** cigarette beetle, *Lasioderma serricorne*, microbial ecology, postharvest, rice weevil, *Sitophilus oryzae*, wheat

## Abstract

Understanding the ability of internal‐ and external‐infesting stored product insects to vector microbes is important for estimating the relative risk that insects pose to postharvest commodities as they move between habitat patches and in the landscape. Thus, the aim of the current study was to evaluate and compare the microbial growth in novel food patches at different dispersal periods by different populations of *Sitophilus oryzae* (e.g., internal‐infesting) and *Lasioderma serricorne* (e.g., external‐infesting). Adults of both species collected from laboratory colonies or field‐captured populations were either placed immediately in a novel food patch, or given a dispersal period of 24 or 72 h in a sterilized environment before entering a surrogate food patch. Vectored microbes in new food patches were imaged after 3 or 5 days of foraging, and microbial growth was processed using ImageJ while fungal species were identified through sequencing the ITS4/5 ribosomal subunit. We found that increasing dispersal time resulted in multiple‐fold reductions in microbial growth surrogate food patches by *L. serricorne* but not *S. oryzae*. This was likely attributable to higher mobility by *S. oryzae* than *L. serricorne*. A total of 20 morphospecies were identified from 13 genera among the 59 sequences, with a total of 23% and 16% classified as *Aspergillus* and *Penicillium* spp. Our data suggest that there is a persistent risk of microbial contamination by both species, which has important food safety implications at food facilities.

## Abstract 2

The study investigated how *Sitophilus oryzae* and *Lasioderma serricorne*, two types of stored product insects (internal‐ and external‐infesting), affect microbial growth in new food patches over varying dispersal periods. We found that longer dispersal times led to a significant reduction in microbial growth in food patches for *L. serricorne* but not for *S. oryzae*, likely due to differences in mobility between the two species. This suggests an ongoing risk of microbial contamination from both insect types, with implications for food safety in storage facilities.

## 1 INTRODUCTION

Insects and microbes represent two of the largest threats to the postharvest supply chain and are responsible for a collective total of over US $100 billion losses annually worldwide (Wacker, 2018). Although microbes and insects can enter the postharvest supply chain at any stage, the carryover of plant pathogens (Shafiekhani & Atungulu, 2017) and some stored product insect species (Tigar et al., 1994) from the field into storage has been partially linked to microbial growth and infestations after harvest at food facilities. While there is this important connection between plant pathogens before and after harvest, there has also been increasing concern globally about food safety. Contamination of stored grain with insect fragments as well as microorganisms remains a major concern for the grain industry as it affects the grains in both quantitative and qualitative losses and the presence of certain microbes can be linked to food safety issues (Yadav et al., 2014). For example, a survey of flour in commercial mills in Italy found viable *Alternaria*, *Penicillium*, *Aspergillus*, and *Fusarium* to be common contaminants (Minutillo et al., 2022). Many of these species can produce harmful mycotoxins and pose serious health risk for consumers (Magan et al., 2003; Ponce et al., 2021). In addition, these fungi have also been found in association with stored product insects (reviewed in Ponce et al., 2021). Although important advances have been made in understanding microbial interactions with insects and plants within each individual agroecosystem (e.g., preharvest and postharvest), much more needs to be done in order to understand the dynamics of these interactions across different agroecosystems.

As a consequence of serving as physical vectors of microbes, stored product insects may influence the production of off‐odors and mycotoxins. Insect activity has been shown to positively influence fungal growth and impact physiology and vice versa. For example, previous research demonstrated that the presence of insects, such as *Sitotroga cerealella* (Olivier) (Lepidoptera: Gelechiidae) on grain kernels provided a favorable environment for the growth of *Fusarium verticillioides*, as well as promoted the production of fumonisin B_1_, an important mycotoxin (Rosa Junior et al., 2019). Likewise, *Fusarium verticillioides* increased progeny production of *Sitophilus zeamais* (Motschulsky) (Coleoptera: Curculionidae). No negative impacts on insect health were noted in this interaction as the fungus was not observed to penetrate the cuticle and the presence of fumonisin B1 extract did not impact fitness (Usseglio et al., 2020). However, in that study, the progeny of *S. zeamais* increased. Furthermore, hotspots of insect infestation may significantly elevate the moisture and temperature in the grain mass, leading to microbial growth and deterioration of durable commodities (Khan et al., 2016). Positive associations were found between the survival rate of *R. dominica* and *S. oryzae* in grain and aflatoxin levels. However, benefits may also accrue to stored product arthropods by the presence of mycoflora on grain. For example, Ponce (2023), Ponce, Sierra, et al. (2023) found that the presence of both *S. oryzae* and *Aspergillus* spp. in a grain mass altered the abiotic conditions in ways that were favorable to both insect species, and it also resulted in elevated progeny production by *S. oryzae* compared to when microbes were absent. As a result, interactions between fungal plant pathogens and stored product insects can sometimes be mutually beneficial.

As mobile organisms, insects are key mediators of plant–microbe, insect–microbe, insect–microbe–plant interactions across multiple agroecosystems because they have the ability to disperse and vector microbes from pre‐ to postharvest environments as well as within a system. In addition, stored product insects can also greatly influence microbial community composition across these different agroecosystems or even within a single agroecosystem. For example, the presence of different species and life stages of stored product insects influence the community composition of microbes (Fandohan et al., 2003; Lamboni & Hell, 2009). Hubert et al. (2021) found that microbes are commonly found in the diet of dust mites; the bacterial genera *Bacillus*, *Staphylococcus*, and *Kocuria* were found in the diet where house dust mites had fed and microbe composition differed depending on the presence of mite life stages, including eggs and mite bodies. The microbial community can be influenced by the ecological succession of stored product insects (and vice versa) (Fandohan et al., 2003; Gerken & Morrison, 2023). Primary or internal‐infesting insect pest will bring their own microbial colonizers to undamaged kernels that may be separate from secondary or external‐infesting pests that can take advantage of damaged kernels and grain dust. The plant pathogens associated with food, *Aspergillus* and *Penicillium* spp., were found in 71% of 988 *L. serricorne* adults captured in traps placed in people's homes (Nakagawa et al., 2008). That study used scanning electron microscopy and found *Aspergillus* spores on the pronotum, forewing, labrum, mandible, scutellum, abdomen, femur, and tibia. For example, the fungal community in bagged coffee was found to shift from being dominated by *Saccharomycetales* during the first 6 months to dominated by *Wallemia* sp. thereafter (Broissin‐Vargas et al., 2018). Thus, common fungi and bacteria, including many plant pathogens seem to be intimately associated with stored product insects.

Microbes can also mediate insect movement and behavior over a range of distances. A recent study demonstrated that fungal volatile cues from *A. flavus* were important for close‐range foraging of *Sitophilus oryzae* (L.) (Coleoptera: Curculionidae), an internal‐infesting stored product pest but they did not influence attraction at longer distances (Ponce et al., 2022). By contrast, for the external‐infesting pest, *Lasioderma serricorne* (L.) (Coleoptera: Ptinidae), fungal cues were important for attraction both at close range and at a distance. More generally, a systematic review by Ponce et al. (2021) found that the behavioral response by the stored product insect community to microbial volatiles and the vectoring capacities of stored product insects may be context and species‐dependent. While a fair bit is known about individuals attracted to habitat patches, less is known about the microbial vectoring status of individuals leaving a patch. This is something which may be passive or active during dispersal within and among food patches, but to what extent and which microbes are vectored, and how insect taxon affects this is mostly unknown. In addition, it is also unclear how dispersal period may affect these patterns. One hypothesis is that as stored product insects disperse, they may dislodge spores from their body and may therefore vector fewer microbes when they arrive at habitats with new commodities with increasing dispersal time. This would have clear management repercussions because insects at the tail end of the dispersal kernel (e.g., the long‐distance dispersers) may then be less risky immigrants when entering food facilities. An alternative hypothesis may be that with increasing dispersal, adults pick up a variety of new spores, making their microbial communities more diverse and abundant when they reach their new food patch, therefore of greater food safety risk. So far as we are aware, neither of these hypotheses has been formally tested with stored product insects, and our goal was to evaluate the first hypothesis.

Monophagous insects may interact with a more limited range of microbes, leading to more, straightforward epidemiological processes and management strategies for both the vector and the microbe. On the other hand, polyphagous species may interact with a diverse suite of microbes, increasing their vectoring potential (Bragard et al., 2019; Chuche & Thiéry, 2014). Since many stored product insects are polyphagous and attack grain under different conditions (e.g., whole and intact vs broken or damaged), understanding the breadth of microbes they can interact with and vector is imperative for improving integrated pest management (IPM) after harvest and reducing risk from pathogens. *Sitophilus oryzae* (L.) (Coleoptera: Curculionidae) and *Lasioderma serricorne* (F.) (Coleoptera: Ptinidae) are two economically significant stored product insects that represent two extremes in life history and interact extensively with microbes in the postharvest environment (Edde, 2019; Hagstrum & Subramanyam, 2006). In addition, *L. serricorne* has been proposed as a model insect for beetle–fungus interactions because of its important symbiosis with the symbiont fungus *Symbiotaphrina* (Martinson, 2020). Likewise, *S. oryzae* has also been found to harbor obligatory endobacterial symbionts, which are required for survival (Kosewska et al., 2023). In addition to endosymbionts, the presence of fungal spores has been documented on the cuticle of *L. serricorne* (Nakagawa et al., 2008). Due to their extensive interactions with a variety of microbes, stored product insects may serve as important physical vectors of microbes, including many with importance to human and animal health. Thus, the aims of the current study were to: (1) evaluate whether stored product insects reliably vector microbes, (2) determine the identity of those microbes through sequencing, (3) understand how insect species may affect vectoring capacity using *L. serricorne* and *S. oryzae* as models, and finally (4) elucidate how dispersal period (0, 24, 72 h) and population origin (colony, field, or those from *A. flavus*‐enriched environments) affect inoculation of new food patches with microbes.

## 2 MATERIALS AND METHODS

### 2.1 Experimental insects and source of grain

Laboratory colonies of *L. serricorne* and *S. oryzae* were obtained from the USDA Agricultural Research Service's (ARS) Center for Grain and Animal Health Research (CGAHR) in Manhattan, KS, USA (referred to hereafter as “colony populations”). Colonies of *S. oryzae* (originally collected from Hudson, Kansas in 2012) were reared on organic whole kernel wheat that tempered to 15% grain moisture from an initial 10.6% moisture with the assistance of a grain moisture meter (DICKEY‐john, GAC2100, Auburn, IL, USA). Colonies with 4‐ to 8‐week‐old adults were kept in 950‐mL mason jars (8.5 D × 17 cm H) and reared at 27.5 ± 0.01°C, 60 ± 3% RH, and 14:10 L:D. Hard whole winter wheat used for rearing and experiments below was obtained from a local Kansas farmer and held in cold storage (~4°C) at the USDA‐ARS Center for Grain and Animal Health Research (CGAHR) for no more than ~3 years until it was used. Immediately prior to use in experiments, wheat was frozen at −4°C for at least 72 h prior to ensure it was not infested by other living arthropods before use in experiments. Laboratory colonies of *L. serricorne* (originally collected from a rice mill in AR in 2012) have been continuously maintained on a diet of 95% bleached wheat flour and 5% brewer's yeast with oats sprinkled on top and a moistened, crumpled towel added. Insects were subcultured by sieving (No. 30 sieve, 594 × 594 um mesh, W.S. Tyler Co., Cleveland, OH, USA) 150 mixed‐sex *L. serricorne* adults and adding them into mason jars (950‐mL capacity) filled two‐thirds of the way to the top with diet. Colonies were maintained at 27.5°C, 65% RH, and 14:10 L:D photoperiod. Individuals were never tested more than once for the experiments below.

### 2.2 Vectoring assay for *Sitophilus oryzae* and *Lasioderma serricorne*

To determine whether colony populations of *L. serricorne* and *S. oryzae* vectored microbes and to identify possible interactions with dispersal time, a vectoring assay was performed for each species. A total of 32 g of potato dextrose agar (Merck, Darmstadt, Germany) was mixed with 900 mL of deionized water in a 1000‐mL glass media bottle with a magnetic stirring rod placed inside the bottle. The agar solution was autoclaved (533LS, Getinge, Rochester, NY, USA) for 30 min and then stirred on a hot plate (Fisher Scientific, Waltham, MA, USA) to slowly cool down for 20 min. Before pouring the agar solution into sterilized Petri dishes (100 × 15 mm W:H), the biosafety cabinet (75 × 73 × 95 cm L:H:W, #302381101, Labconco, Kansas City, MO, USA) was sanitized with 70% ethanol and exposed to ultraviolet light for 10 min. A total of ~30 Petri dishes with potato dextrose agar were allowed to solidify overnight for ~12 h. Fresh Petri dishes were used for each experimental replicate.

For the vectoring assay, the impact of dispersal (0, 24, or 72 h) and foraging time (3 or 5 days) on vectoring ability were tested. Briefly, adult *L. serricorne* or *S. oryzae* were singly removed from colony containers with sterilized forceps and then placed immediately in the center of Petri dish containing PDA for the 0 h dispersal period. Alternatively, some insects were given a 24 or 72 h dispersal period in an autoclaved 4 L‐capacity glass container and stored at constant conditions of 25°C, 60% RH, and 14:10 L:D photoperiod prior to being added to the PDA. Petri dishes were maintained at 30°C, 60% RH, and 14:10 L:D photoperiod for either 3 or 5 days, then photographed for microbial growth. Each novel food patch consisted of an agar Petri dish sealed with parafilm and stored in an environmental chamber. In a second round of testing, adult *L. serricorne* or *S. oryzae* dispersed in much smaller autoclaved 950‐mL glass mason jars for 24 and 72 h prior to transferring them to agar dishes. To further avoid contaminants in the second round, all glass mason jars were first cleaned with 70% ethanol and then sanitized with ultraviolet light for 10 min in a walk‐in environmental chamber prior to placing single adults in each. Transfer of *L. serricorne* or *S. oryzae* adults from dispersal containers to agar at the conclusion of the dispersal period was performed inside the biosafety cabinet to prevent contamination of dishes.

Pictures of the agar dishes and corresponding microbial growth were taken using a DSLR camera (EOS 7D Mark II, Canon, Tokyo, Japan) mounted to 3D imaging StackShot (CogniSys, Inc., Traverse City, MI, USA) equipped with a dual flash (MT‐26EX‐RT, Canon, Tokyo, Japan). Light was diffused using a partially cut frosted plastic jar (15.2 × 7.6 cm D:H) making a total of *n* = 60 replicates per treatment combination (of dispersal time, insect species, and foraging time in patch). The pictures taken were processed using ImageJ 1.53a (Wayne Rasband, National Institutes of Health, USA) to quantify the microbial growth in the agar dishes. The images had their backgrounds subtracted and then were processed using the *find edges* tool. Finally, they were converted to binary and either dilated or eroded to conform to the original image parameters. A circle encompassing the Petri dish was created and the mean grayscale, standard deviation of the grayscale value, and count of pixels were measured as a surrogate for microbial growth on the dishes. This allowed a quantitative measure of microbial growth by creating an average in a given image. The mean grayscale value could range from 0 (full white), indicating no microbial growth to 255 (full black), indicating full microbial growth on the entire dish. Finally, visually, microbial morphospecies (alpha) richness was assigned to each image given the number of unique morphospecies on the plate as a proxy for community complexity.

### 2.3 Individuals from microbially enriched environments for vectoring assay

In order to determine whether high microbial load from an initial food patch can affect subsequent vectoring patterns, we examined the vectoring capacity of *S. oryzae* exposed to grain inoculated with *A. flavus* (e.g., AF, hereafter). To prepare the AF, 600 g of grain was added to a stainless‐steel pot filled with water and placed on a hot plate at 500°C. Once boiling for 15 min, the water was drained and the grain was evenly spread out on sterile wipes (38.1 × 42.5 cm, 3 ply, Tech wipes, Skilcraft, NIB, Alexandria, VA) and allowed to dry inside a laminar fume hood (ca. 3 h). Afterward, the grain was evenly divided (~300 g) and placed in two separate autoclaved mason jars (950‐mL capacity). A single hole was pierced through each lid and lined with a cotton ball. The jars were then sealed with aluminum foil and were autoclaved (533LS, Getinge, Rochester, NY, USA) for 30 min. To inoculate with *A. flavus*, a 3‐inch strip of agar containing a pure culture of *A. flavus* grown on PDA for 7 days at 30°C, 60% RH, and 14:10 L:D photoperiod (Ponce, 2023; Ponce, Sierra, et al., 2023) was placed into each jar containing the grain. AF was then maintained at room temperature for roughly 10 days or until the *A. flavus* evenly covered as much of the grain as possible. Batches of inoculated grain were used within 10–15 days of preparation. Grain was never used more than once for each replicate of every trial in each assay experiment to prevent cross‐contamination. A total of 75 insects were added to 300 g AF in a 950‐mL mason jar and allowed to forage for 2 weeks prior to use in the vectoring experiment. The same dispersal periods (0, 24, 72 h) and time in patch (3 and 5 days) described above were used for this experiment. The mean grayscale value and microbial morphospecies richness were recorded for each image. There were a total of *n* = 30 replicates per treatment combination.

### 2.4 Field‐collected individuals for vectoring assay

To assess how the species richness and microbial growth of the vectored community differed between colony‐reared and field‐collected individuals laboratory adults, *L. serricorne* and *S. oryzae* individuals were also collected from the field. Due to their relatively short lifespan in captivity and difficulty in procuring live adults, field‐caught individuals were only assessed after a dispersal period of 0 h. To obtain sufficient numbers of adults, insects were caught at four different field sites around the area of greater Manhattan, KS including (1) a site with a preharvest wheat field bordered by woodlands (39°14′26.2″ N, 96°34′59.1″ W), (2) local apartment complex consisting of end consumers (39°11′43.6″N, 96°36′07.4″W), (3) Kansas State University Agronomy Farm with storage silos (39°12′23.7″N, 96°35′43.2″W), and (4) a private residence adjacent to a working cattle farm (39°12′23.7″N, 96°35′43.2″W). In each location, a total of three 4‐funnel Lindgren traps (Bioquip, Rancho Dominguez, CA, USA) were deployed at least 10 m apart at about 1 m height on rebar or hung from a tree along the perimeter of the location site and were baited with a multispecies lure containing both *L. serricorne* sex pheromone and *Sitophilus* spp. pheromone (PTL bullet lure, #IL‐108, and *Sitophilus* spp. bullet lure, #IL‐703, Insects Limited, Westfield, IN, USA). In addition, three ground traps were deployed that consisted of commercially available pitfall traps (Dome®, Storgard, Trécé, Adair, OK, USA) with two connectible pieces (Doud et al., 2021; Doud & Phillips, 2020), containing a central well where a *Sitophilus* spp. lure was added along with a 5 g of whole maize as a kairomone bait. Pheromone lures were changed every 60 days. No kill mechanism was added because adults needed to be alive. Traps were checked on a daily basis for capture of new adults and brought immediately back into the laboratory in separate unused, sterilized containers for addition to agar dishes. Stored product insects were identified using taxonomic keys in USDA (1991). Dispersal period at 0 h and time in patch (3 and 5 days) as described above were used for this experiment. The mean grayscale value and microbial morphospecies richness were recorded for each image. There were a total of *n* = 30 replicates per treatment combination.

### 2.5 Microbial morphotype sequencing

To identify microbes associated with the cuticles of colony‐reared and field‐caught *L. serricorne* and *S. oryzae*, unique fungal morphotypes were isolated from cuticles and cultured through the vectoring assay for the purpose of sequencing. Pure isolations from the unique morphotypes were made by excising a 1 × 1 cm agar plug of fungi and subculturing it onto a new potato dextrose agar dish, sealed with parafilm to obtain a pure culture. This was sometimes done multiple times until pure isolations were obtained. DNA was extracted from pure cultures after 7 days of growth in an environmental chamber set to 30°C, 60% RH, and 14:10 L:D photoperiod using the Quick‐DNA Fecal/Soil Microbe Miniprep Kit (D6010, Zymo Research Corp, Irvine, CA, USA). The concentration of DNA and quality were assessed using the Take 3 Assay on a microplate reader (Gen5™, BioTek Instruments, Winooski, VT, USA) before performing the polymerase chain reaction (PCR).

Polymerase chain reaction was used to amplify the internal transcribed spacer (ITS) region for each morphotype using primer sets: ITS4 5′‐TCCTCCGCTTATTGATATGC‐3′ and ITS5 5′‐GGAAGTAAAAGTCGTAACAAGG‐3′ (White et al., 1990). To facilitate species‐level identification for *Fusarium* spp., the translation elongation factor 1 alpha (TEF1α) region and the RNA polymerase II subunit (RBP2) region were also amplified with the following respective primer pairs: EF1 (5′‐ATGGGTAAGGARGACAAGAC‐3′)/EF2 (5′‐GGARGTACCAGTSATCATGTT‐3′) and RBP2‐5F2 (5′‐GGGGWGAYCAGAAGAAGGC‐3′)/RPB2‐7cR (5′‐CCCATRGCTTGYTTRCCCAT‐3′) (LeBlanc et al., 2015; O'Donnell et al., 1998, 2007). Each reaction contained 1.0 μL extracted DNA, 1.0 μL of each primer (10 μM), 9.5 μL of nuclease‐free water, and 12.5 μL of master mix containing 50 units/mL of Taq DNA polymerase master mix (Hot Start Taq 2X Master Mix, Promega, Madison, WI, USA). Briefly, the PCR program consisted of 2 min of initial denaturation at 95°C, followed by 30 cycles of 95°C for 30 s, 55°C for 1 min, and 72°C for 1.5 min, and a final extension at 72°C for 5 min (S1000 Thermal Cycler, Bio‐Rad, Hercules, CA, USA). PCR products were treated with ExoSAP‐IT prior to sequencing following the manufacturer's protocol (ThermoFisher Scientific, Waltham, MA, USA). Amplicons were sequenced bidirectionally on an ABI 3730XL instrument (Eurofins Scientific, Brussels, Belgium), and the resulting sequences were quality‐filtered and aligned using Geneious Prime 2021.0.3 (Biomatters Ltd, Auckland, New Zealand).

The consensus sequences were searched against NCBI's nucleotide database (nt) using the BLASTn algorithm (Altschul et al., 1997). In order to circumvent taxonomic misassignments, the ITS consensus sequences were also checked against the UNITE Database using the Ribosomal Database Project Classifier algorithm (Wang et al., 2007). The consensus sequence for the ITS sequence of *A. flavus* was submitted to GenBank under OM490684 while the ITS, TEF1α, and RBP2 sequences from *F. verticillioides* were deposited under OM460744, OM542207, and OM542208. The morphospecies richness sequences were deposited under MMXXXXXX–ZZXXXXXX.

### 2.6 Statistical analysis

A linear mixed model was used to examine the microbial growth (mean grayscale value) in agar dishes (e.g., novel food patches), with separate models by population. Dispersal period (0, 24, or 72 h), time in patch (3 or 5 days), and species (*L. serricorne* or *S. oryzae*) were used as fixed, explanatory variables in the model. Date was a random blocking variable. An initial model was created without interactions to inspect residuals and diagnostic plots, and if there were deviations from normality or homoscedasticity, a log transformation was used where appropriate, which corrected the problem. Upon a significant result from the overall model, Tukey HSD was used for multiple comparisons. For this and all other tests, R Software was used (R Core Team, 2022), with *α* = 0.05.

In addition, a linear mixed model was used to analyze morphotype richness in agar dishes, with separate models by population, and using the same model form as above. Dispersal period (0, 24, or 72 h), time in patch (3 or 5 days), and species (*L. serricorne* or *S. oryzae*) were used as fixed, explanatory variables in the model. Date was a random blocking variable. Residuals were inspected, and datasets with deviations from assumptions were log‐transformed. Tukey HSD was used for multiple comparisons upon a significant result from the model.

## 3 RESULTS

### 3.1 Effects of dispersal on *Lasioderma serricorne* and *Sitophilus oryzae*

**FIGURE 1:**
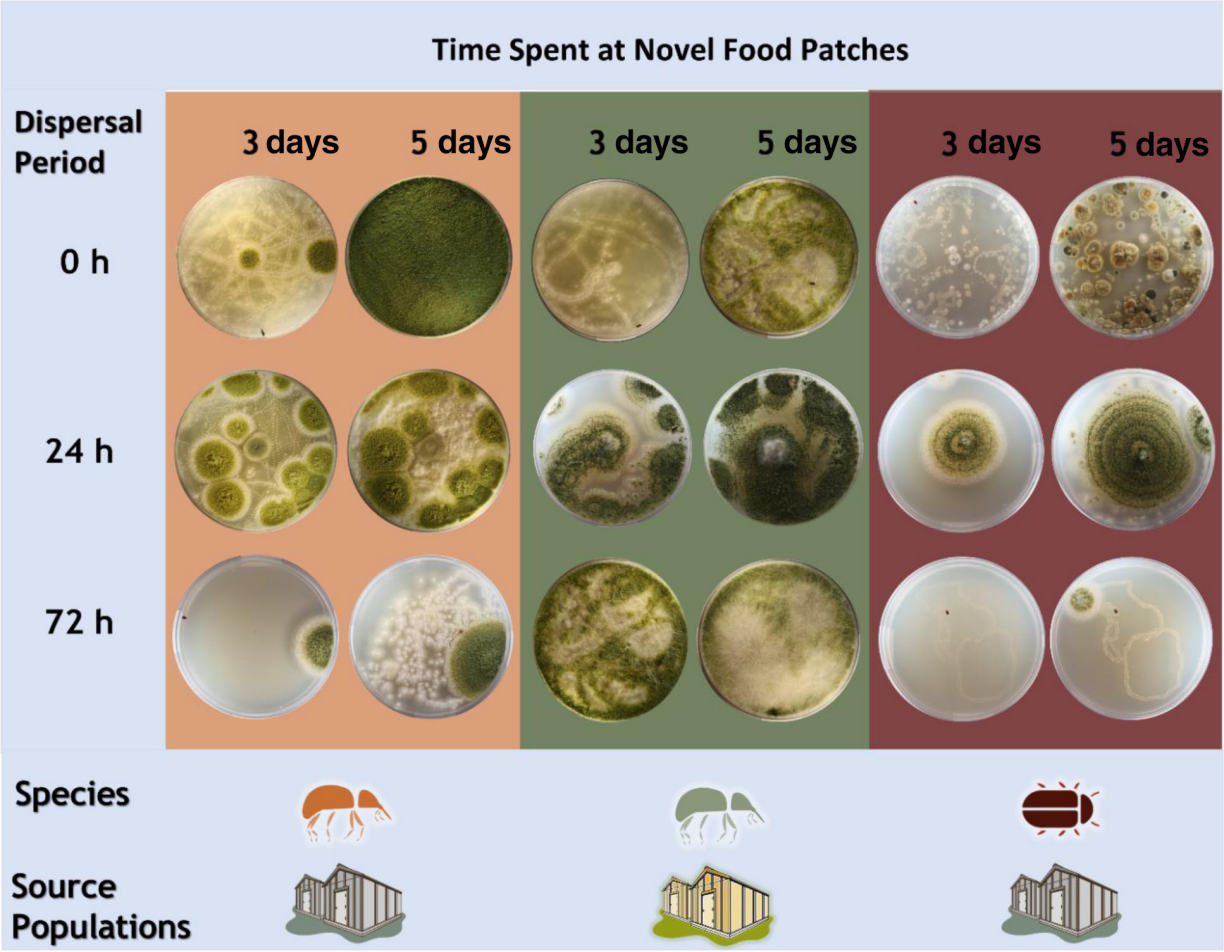
Habitus images of factitious foraging patches from the vectoring assay showing that microbes are transmitted physically by *Sitophilus oryzae* (left orange and green boxes) and *Lasioderma serricorne*, (right red box) but at decreasing frequency and diversity with increasing dispersal periods (0, 24, 72 h) prior to entering a foraging patch. *S. oryzae* originally from laboratory colonies (orange box) or *Aspergillus flavus*‐inoculated grain (e.g., microbially enriched environments; green box). The longer that *S. oryzae* and *L. serricorne* were in foraging patches (3 vs. 5 days), the greater the microbial growth under constant conditions (30°C, 60% RH, and 14:10 L:D photoperiod). Pictures are representative images taken from a total of *n* = 30 replicates for each dispersal period and foraging time in a food patch.

**FIGURE 2:**
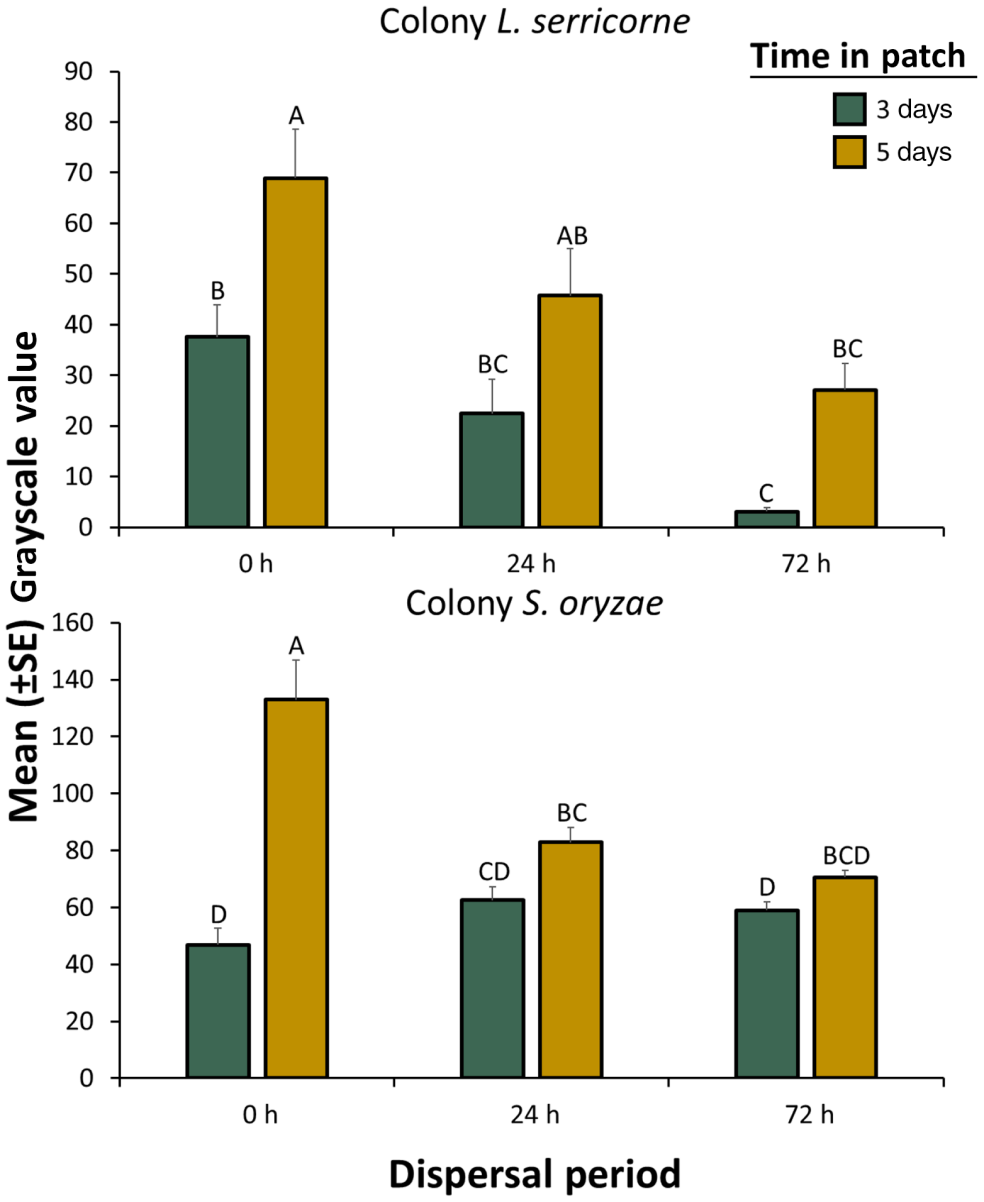
Mean grayscale value (±SE, e.g., surrogate measure of microbial growth) for microbes vectored by *Lasioderma serricorne* (top panel) and *Sitophilus oryzae* (bottom panel) adults from the colony population after they were given a dispersal period of 0, 24, or 72 h and then placed in factitious foraging patches for 3 days (green bars) or 5 days (yellow bars). There were a total of *n* = 30 replicate adults tested per bar. Bars with shared letters are not significantly different from each other (Tukey HSD, *α* = 0.05). The grayscale value can range from 0, indicating no microbial growth, to 255, indicating the full agar dish was covered with microbes. Error bars represent standard errors.

For *L. serricorne*, initial microbial growth at 3 days decreased significantly by 92% as dispersal period increased from 0 to 72 h (Table 1, Figure 1). Similar results were shown for total microbial growth after 5 days in the food patch, with a 43% decrease as dispersal period increased from 0 to 72 h (Figure 2). By contrast, for *S. oryzae*, initial microbial growth at 3 days did not change as dispersal period increased (Table 1) while the total microbial growth after 5 days decreased from 0 to 24 h but then stabilized after 72 h (Tukey HSD, Figure 2). This was also corroborated visually (Figure 1). Clear trails of microbes from the movement of both species were found in the novel food patches with tarsal imprints apparent (Figure 1). Across dispersal periods, microbial inoculation of new environments quickly proceeded with time spent in patch for *L. serricorne* and *S. oryzae* (Table 1), with 2.2‐ and 1.7‐fold, respectively, more microbial growth at 5 days than at 3 days (Figure 2). Bacteria and/or yeast appeared to dominate at 3 days while other fungi dominated by 5 days. While the interaction between dispersal period and time in patch significantly affected microbial growth for *S. oryzae*, there was no interaction for *L. serricorne* (Table 1).

**TABLE 1:** Summary of ANOVA models for mean grayscale value (microbial growth) and morphotype richness after the introduction of *L. serricorne* or *S. oryzae* on PDA agar dishes after 0, 24, or 72 h dispersal from laboratory colonies.

Variable	Mean grayscale value			Species richness	
	df	*F*	*p*	*F*	*p*
*L. serricorne*					
Dispersal period	2	6.05	.003	2.26	.11
Time in patch	1	10.0	.002	22.0	.0001
Interaction	2	0.20	.82	11.4	.0001
Residuals	174				
*S. oryzae*					
Dispersal period	2	1.39	.25	39.4	.0001
Time in patch	1	76.6	.0001	140	.0001
Interaction	2	17.1	.0001	33.0	.0001
Residuals	174				

Abbreviations: *L. serricorne*, *Lasioderma serricorne*; *S. oryzae*, *Sitophilus oryzae*.

**FIGURE 3:**
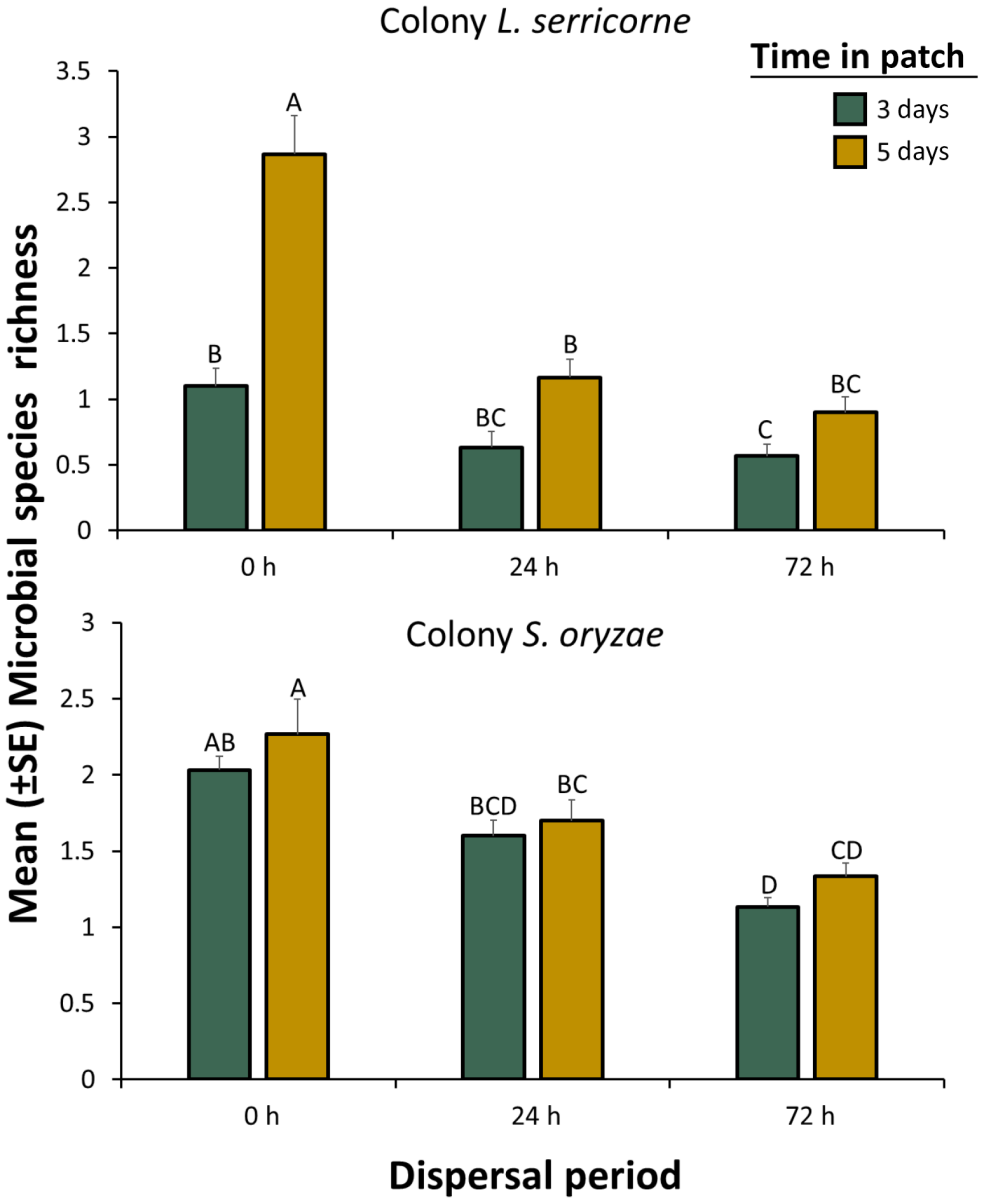
Mean species richness (±SE) for microbes vectored by *Lasioderma serricorne* (top panel) and *Sitophilus oryzae* (bottom panel) adults from the colony population after they were given a dispersal period of 0, 24, or 72 h and then placed in factitious foraging patches for 3 days (green bars) or 5 days (yellow bars). There were a total of *n* = 30 replicate adults tested per bar. Bars with shared letters are not significantly different from each other (Tukey HSD, *α* = 0.05). Species richness represents the number of microbial morphotypes in each food patch. Error bars represent standard errors.

In evaluating the morphotype richness vectored by *L. serricorne*, there was no significant difference between the two dispersal periods (Table 1). Unlike *L. serricorne*, *S. oryzae* showed a significant reduction in morphotype richness with increasing dispersal period (Table 1), with a 43% reduction in morphotype richness from 0 to 72 h (Figure 3). Time in patch significantly affected morphotype richness of microbes vectored by *L. serricorne* and *S. oryzae*, with 2.1‐ and 1.1‐fold, respectively, relatively more species vectored at 5 days compared to 3 days (Figure 3). There were significant two‐way interactions between dispersal period and time in patch that affected morphotype richness for *L. serricorne* and *S. oryzae*. For example, the morphotype richness of microbes vectored by *L. serricorne* was reduced by almost 70% at 5 days in the food patch when comparing the 0 h to a 72 h dispersal period, but the morphotype richness was only reduced by 48% at 3 days in patch between the two periods (Figure 3).

### 3.2 Effects of dispersal from a microbially enriched environment by *Sitophilus oryzae*

**FIGURE 4:**
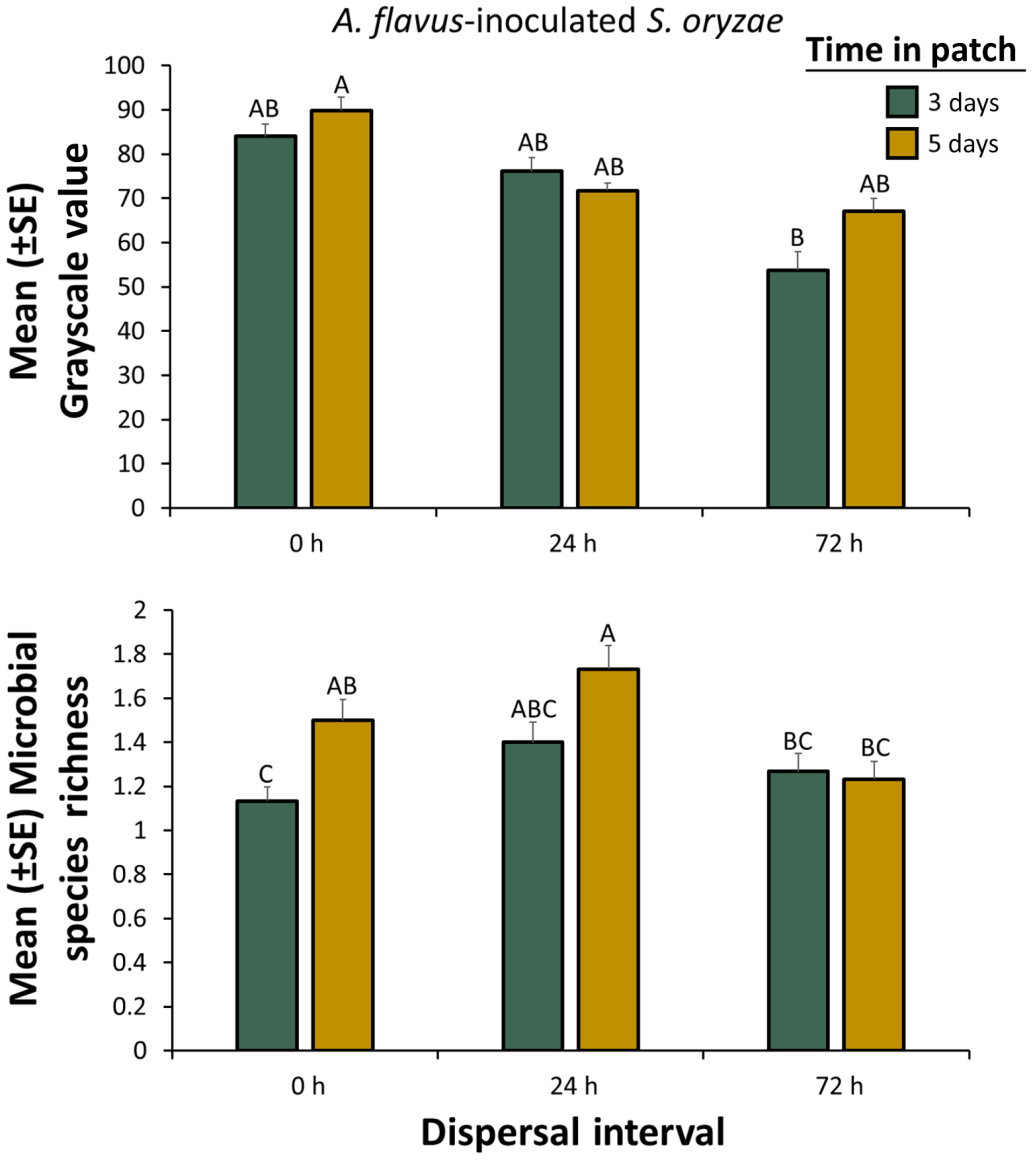
Mean grayscale value (e.g., surrogate measure of microbial growth; top panel) or mean species richness (e.g., surrogate measure of microbial diversity; bottom panel) for microbes vectored by *Sitophilus oryzae* from *Aspergillus flavus*‐inoculated grain after they were given a dispersal period of 0, 24, or 72 h and then placed in factitious foraging patches for 3 days (green bars) or 5 days (yellow bars). There were a total of *n* = 30 replicate adults tested per bar. Bars with shared letters are not significantly different from each other (Tukey HSD, *α* = 0.05). The grayscale value can range from 0, indicating no microbial growth, to 255, indicating the full agar dish was covered with microbes. Species richness represents the number of microbial morphotypes in each food patch. Error bars represent standard errors.

For individuals dispersing from microbially rich environments, dispersal period significantly affected microbial growth of vectored microbes by *S. oryzae* (Table 2). In particular, microbial growth was reduced by 31% on average after *S. oryzae* dispersal increased from a 0 to 72 h across time in patch (Figure 4). Time in patch did not significantly change microbial growth in the *S. oryzae* treatment (Table 2) because it remained relatively stable after a 24 h dispersal period between 71 and 76 (mean grayscale) at 3–5 days (Tukey HSD, Figure 4). Nonetheless, there was a significant interaction between dispersal period and time in patch on the microbial growth induced by *S. oryzae* (Table 2), but this was a quantitative interaction, with no change in the effect direction.

**TABLE 2:** Summary of ANOVA models for mean grayscale value (microbial growth) and morphotype richness after the introduction of *S. oryzae* on PDA agar dishes after 0, 24, or 72 h dispersal from grain inoculated with *A. flavus*.

Variable	Mean grayscale value			Species richness	
	df	*F*	*p*	*F*	*p*
*S. oryzae*					
Dispersal period	2	26.7	.0001	28.1	.0001
Time in patch	1	1.87	.17	38.2	.0001
Interaction	2	4.20	.02	13.1	.0001
Residuals	174				

Abbreviations: *A. flavus*; *Aspergillus flavus*; *S. oryzae*, *Sitophilus oryzae*.

In terms of morphotype richness, dispersal period had a significant impact on *S. oryzae* when they were reared in microbially rich environments after they spent 5 days in patch (Table 2). There were 1.2–1.3‐fold more morphotypes vectored by *S. oryzae* after a 24 h dispersal period compared to 0 or 72 h, respectively (Figure 4). In addition, overall the time in patch significantly affected the morphotype richness of microbes vectored by *S. oryzae* (Table 2). On average, there were 1.2‐fold more morphotypes vectored by *S. oryzae* at 5 days compared to 3 days. Finally, there was also a significant interaction between dispersal period and time in patch on morphotype richness of microbes vectored by *S. oryzae*, with a 29% decrease in morphospecies diversity at 72 h compared to 24 h after 5 days in patch.

### 3.3 Effects of population origin on vectoring capacity

**FIGURE 5:**
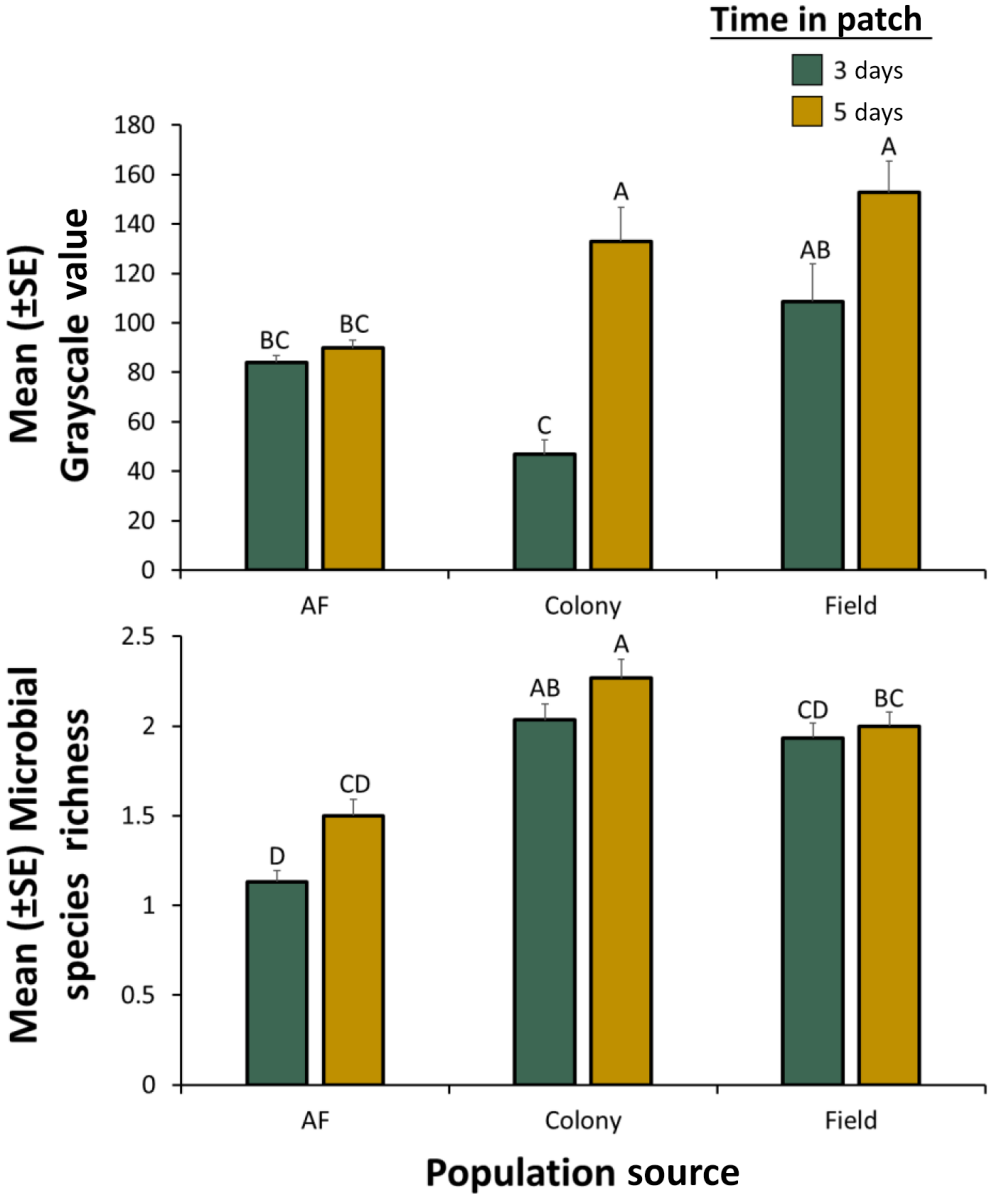
Mean grayscale value (e.g., surrogate measure of microbial growth; top panel) or mean species richness (e.g., surrogate measure of microbial diversity; bottom panel) for microbes vectored by *Sitophilus oryzae*, depending on population source (from *Aspergillus flavus*‐inoculated grain, AF; laboratory colonies, Colony; or field‐captured adults, Field) after they were given a dispersal period of 0 h and then placed in factitious foraging patches for 3 days (green bars) or 5 days (yellow bars). There were a total of *n* = 30 replicate adults tested per bar. Bars with shared letters are not significantly different from each other (Tukey HSD, *α* = 0.05). The grayscale value can range from 0, indicating no microbial growth, to 255, indicating the full agar dish was covered with microbes. Species richness represents the number of microbial morphotypes in each food patch. Error bars represent standard errors.

Time in patch by *S. oryzae* did not have a significant effect on microbial growth (Table 3). However, population origin significantly affected microbial growth by dispersing *S. oryzae* (Figure 5, Table 3). There was 1.7–1.8‐fold more microbial growth from field populations of stored product insects than *S. oryzae* from laboratory colonies or *A. flavus*‐inoculated grain, respectively, regardless of time in patch.

**TABLE 3:** Summary of ANOVA models for mean grayscale value (microbial growth) and morphotype richness after the introduction of *S. oryzae* from different populations (colony, *A. flavus*‐inoculated grain, and field) on PDA agar dishes after a 0 h dispersal period.

Variable	Mean grayscale value			Species richness	
	df	*F*	*p*	*F*	*p*
*S. oryzae*					
Time in patch	1	0.93	.33	18.8	.0001
Population origin	2	22.6	.0001	8.44	.001
Time in patch:Population origin	2	1.25	.26	1.84	.16
Residuals	196				

Abbreviations: *A. flavus*; *Aspergillus flavus*; *S. oryzae*, *Sitophilus oryzae*.

Moreover, time in patch significantly affected morphospecies richness, with 3.5, 9.0, and 25% greater morphotype richness at 5 days compared to 3 days for the field, colony, and AF‐inoculated population of *S. oryzae*, respectively. Population origin also significantly affected morphotype richness by dispersing *S. oryzae* (Table 3). There was 1.3–1.7‐fold greater morphospecies richness from field populations of stored product insects than *S. oryzae* from *A. flavus*‐inoculated grain, respectively (Figure 5). The colony populations had 1.05–1.14‐fold greater morphospecies richness than field populations of *S. oryzae*.

### 3.4 Effects of species on vectoring capacity

Stored product beetle species significantly affected microbial growth in novel factitious food patches (Table S1), with 2.3‐fold more microbial growth in patches with *S. oryzae* than *L. serricorne* (Figure S1). There was also a significant two‐way interaction between time in patch and beetle species (Table S1). However, this was only a quantitative interaction, with no change in the direction of the effect size. At 5 days, *S. oryzae* and *L. serricorne* had created 1.7–2.2‐fold more microbial growth than at 3 days in the food patches. Finally, there was also a significant three‐way interaction among all the variables in the model (Table S1). Apparent microbial load was greater on *S. oryzae* than *L. serricorne* at all‐time points.

Microbial morphotype richness of vectored microbes was significantly affected by stored product beetle species in food patch (Table S1), with 1.4‐fold greater species richness when *S. oryzae* was present compared to *L. serricorne* across other treatments (Figure S2). There was a significant interaction between beetle species and dispersal period but not beetle species and time in patch on microbial morphotype richness (Table S1, Figure S2). In particular, there was no significant difference in the morphotype richness of microbes between the two beetle species at 0 h dispersal, but by 24 and 72 h dispersal period, *L. serricorne* appeared to more quickly suffer a loss in the morphotype richness of microbes being vectored compared to *S. oryzae* (Figure S2). Finally, there was a significant three‐way interaction among all the variables (Table S1) on morphotype richness of microbes being vectored.

### 3.5 Community composition of microbes through sequencing

**FIGURE 6:**
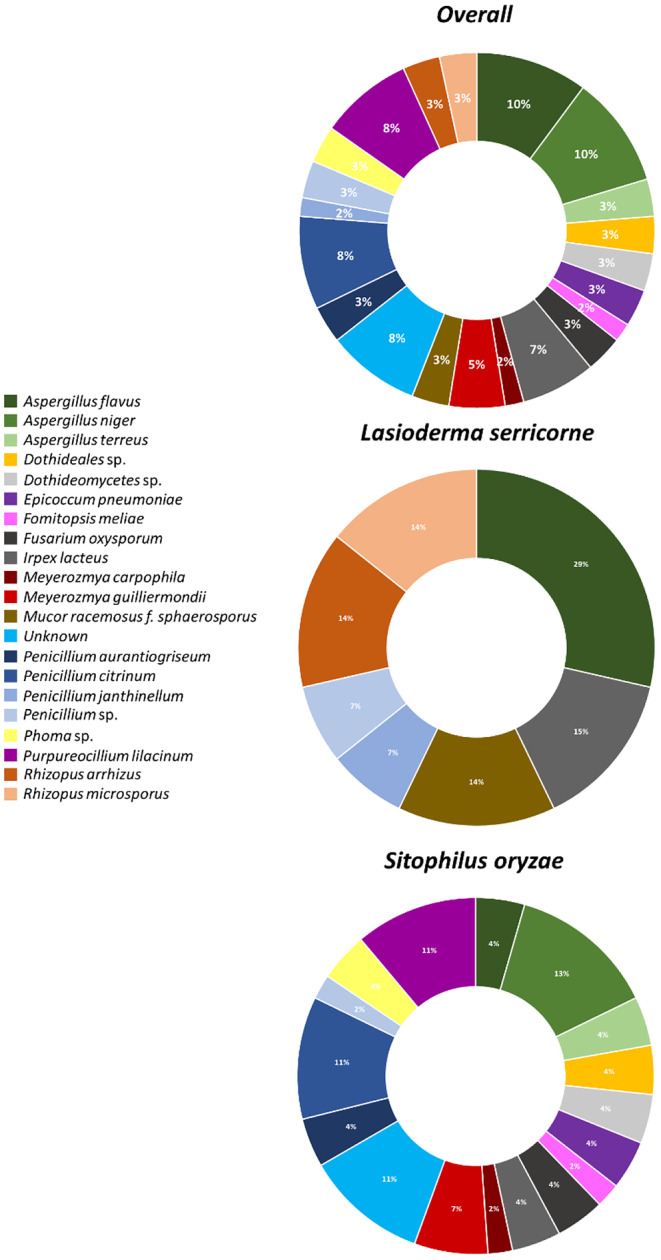
Community composition of microbes derived via sequencing the ITS4/5 ribosomal subunit from environmental fungi vectored and dispersed on PDA dishes by *Sitophilus oryzae* and *Lasioderma serricorne*. Percentages displayed for each taxon are portrayed in the circle. *Aspergillus* spp. comprised over half of the samples.

In total, there were 20 microbe species identified and 13 genera were collected from 59 sequences (Table 4, Figure 6). A total of 23% of the sequences contained *Aspergillus* spp., while 16% contained *Penicillium* spp. The next two most abundant taxa were *Fomitopsis meliae* (8%) and *Meyerozmya* spp. (7%), and *Irpex lacteus* (7%). The remaining taxa comprised 2%–3% of the total samples (Figure 6).

**TABLE 4:** Summary of microbial identifications from each morphotype via sequencing the ribosomal ITS5/4 subunit.

Name	Source population	Species^a^	Identification	Percent identity (%)	Query cover (%)	Accession
IS_WC_EF12_S1_EF1	Lab	RW	*Purpureocullium lilacinum*	99.87	96	MK503783.1
IS_WC_ITS45_S1_ITS4	Lab	RW	*Purpureocillium lilacinum*	99.66	98	JQ863231.1
IS_WC_ITS45_S1_ITS5	Lab	RW	*Purpureocillium lilacinum*	99.66	98	JQ863231.1
IS_WC_ITS45_S2_ITS4	Lab	RW	*Penicillium citrinum*	99.63	97	OR346130.1
IS_WC_ITS45_S2_ITS5	Lab	RW	*Penicillium citrinum*	99.82	98	LC514694.1
IS_WC_ITS45_S3_ITS4	Lab	RW	*Fusarium oxysporum*	99.62	98	MG661728.1
IS_WC_ITS45_S3_ITS5	Lab	RW	*Fusarium oxysporum*	99.81	97	MN909341.1
IS_WC_ITS45_S4_ITS4	Lab	RW	*Meyerozyma carpophila*	99.5	98	MN997029.1
IS_WC_ITS45_S4_ITS5	Lab	RW	*Meyerozyma guilliermondii*	100	97	EF222224.1
IS_WC_ITS45_S5_ITS4	Lab	RW	*Penicillium citrinum*	100	98	MT875277.1
IS_WC_ITS45_S5_ITS5	Lab	RW	*Penicillium* sp.	99.27	98	MK246010.1
IS_WC_ITS45_S7_ITS4	Lab	RW	*Fomitopsis meliae*	99.69	98	KC585351.1
IS_WC_ITS45_S7_ITS5	Lab	RW	n/a	n/a	n/a	n/a
IS_WC_ITS45_S8_ITS4	Lab	RW	*Meyerozyma guilliermondii*	99.50	98	DQ683005.1
IS_WC_ITS45_S8_ITS5	Lab	RW	*Meyerozyma guilliermondii*	99.66	98	LC422339.1
IS_WC_ITS45_S9_ITS4	Lab	RW	n/a	n/a	n/a	n/a
IS_WC_ITS45_S9_ITS5	Lab	RW	n/a	n/a	n/a	n/a
IS_WC_ITS45_S10_ITS4	Lab	RW	*Penicillium aurantiogriseum*	99.31	98	KY552626.1
IS_WC_ITS45_S10_ITS5	Lab	RW	*Penicillium aurantiogriseum*	99.82	97	OQ457008.1
IS_WC_NL41_S1_NL1	Lab	RW	*Purpureocullium lilacinum*	95.28	98	MT453285.1
IS_WC_NL41_S1_NL4	Lab	RW	*Purpureocullium lilacinum*	95.28	98	MT453285.1
IS_WC_NL41_S2_NL1	Lab	RW	*Penicillium citrinum*	95.92	98	LT558885.1
IS_WC_NL41_S2_NL4	Lab	RW	*Penicillium citrinum*	99.30	97	MH990629.1
IS_WC_RBPFR_S6_RBP25F2	Lab	RW	n/a	n/a	n/a	n/a
IS_WC_RBPFR_S6_RBP27CR	Lab	RW	n/a	n/a	n/a	n/a
MP_CBF_ITS45_F2_ITS4	Field	F	*Irpex lacteus*	99.69	96	KJ093494.1
MP_CBF_ITS45_F2_ITS5	Field	F	*Irpex lacteus*	99.69	97	MT276332.1
MP_CBF_ITS45_F6_ITS4	Field	F	*Penicillium janthinellum*	99.66	98	KM268715.1
MP_CBF_ITS45_F6_ITS5	Field	F	*Penicillium* sp.	99.48	97	MG827172.1
MP_CBF_ITS45_F8_ITS4	Field	F	*Rhizopus microsporus*	98.82	97	MG250464.1
MP_CBF_ITS45_F8_ITS5	Field	F	*Rhizopus microsporus*	99.7‐	96	MH473977.1
MP_CBF_ITS45_F9_ITS4	Field	F	*Mucor racemosus f. sphaerosporus*	92.80	98	MK078595.1
MP_CBF_ITS45_F9_ITS5	Field	F	*Mucor racemosus f. sphaerosporus*	93.15	96	MH859527.1
MP_CBF_ITS45_F16_ITS4	Field	F	*Rhizopus arrhizus*	99.51	96	MN547407.1
MP_CBF_ITS45_F16_ITS5	Field	F	*Rhizopus arrhizus*	99.51	97	JQ683247.1
MP_CBF_ITS45_L1_ITS4	Field	F	*Aspergillus flavus*	99.66	98	MK108380.1
MP_CBF_ITS45_L1_ITS5	Field	F	*Aspergillus flavus*	99.66	98	MW709416.1
MP_CBF_NL41_F10_NL1	Field	F	*Aspergillus flavus*	98.86	99	CP051065.1
MP_CBF_NL41_F10_NL4	Field	F	*Aspergillus flavus*	98.69	95	CP051065.1
MP_WF_EF12_F23_EF1	Field	W	*Aspergillus niger*	98.50	95	AM270408.1
MP_WF_EF12_F23_EF2	Field	W	*Aspergillus niger*	98.75	95	AM270408.1
MP_WF_ITS45_F2_ITS4	Field	W	Irpex lacteus	100	96	MF161243.1
MP_WF_ITS45_F2_ITS5	Field	W	Irpex lacteus	99.54	99	MK343588.1
MP_WF_ITS45_F9_ITS4	Field	W	*Aspergillus flavus*	99.49	98	MK461562.1
MP_WF_ITS45_F9_ITS5	Field	W	*Aspergillus flavus*	99.83	96	MW789017.1
MP_WF_ITS45_F22_ITS4	Field	W	*Aspergillus terreus*	99.83	97	MT141149.1
MP_WF_ITS45_F22_ITS5	Field	W	*Aspergillus terreus*	100	97	MW789020.1
MP_WF_ITS45_F23_ITS4	Field	W	*Aspergillus niger*	99.33	98	MN069568.1
MP_WF_ITS45_F23_ITS5	Field	W	*Aspergillus niger*	100	96	KP131604.1
MP_WF_ITS45_F27_ITS4	Field	W	*Dothideomycetes* sp.	100	96	OQ673567.1
MP_WF_ITS45_F27_ITS5	Field	W	*Dothideomycetes* sp.	100	96	KM979499.1
MP_WF_ITS45_F30_ITS4	Field	W	*Dothideales* sp.	98.66	97	MF435051.1
MP_WF_ITS45_F30_ITS5	Field	W	*Dothideales* sp.	100	96	OQ673564.1
MP_WF_NL41_F23_NL1	Field	W	*Aspergillus niger*	98.51	97	KJ365316.1
MP_WF_NL41_F23_NL4	Field	W	*Aspergillus niger*	97.75	99	KJ365316.1
MP_WF_NL41_F30_NL1	Field	W	*Phoma* sp.	98.29	97	KT462714.1
MP_WF_NL41_F30_NL4	Field	W	*Phoma* sp.	99.63	98	KT462714.1
MP_WF_RBPFR_F30_RBP25F2	Field	W	*Epicoccum pneumoniae*	99.78	95	LT593100.1
MP_WF_RBPFR_F30_RBP27CR	Field	W	*Epicoccum pneumoniae*	99.89	95	LT593100.1

^a^ CB—cigarette beetle, *L. serricorne*, F—field‐collected stored product insects, either *L. serricorne* or *T. variabile*, W—weevil, either *S. oryzae* or *S. zeamais*, RW—rice weevil, *S. oryzae*. According to keys in USDA (1991).

There were only six morphotypes identified from *L. serrcorne* from 14 sequenced samples and 29% of them were *Aspergillus flavus*. This was followed by 28% of the sequenced samples consisting of *Rhizopus* spp. (Figure 6).

By contrast, there were many more morphotypes identified from *S. orzyae*, including 15 species from 45 sequenced samples. In total 21% and 17% of the samples belonged to *Aspergillus* spp. and *Penicillium* spp., respectively (Figure 6).

## 4 DISCUSSION

Stored product insects readily disperse from the landscape to food facilities and vice versa, sometimes over extended distances (Campbell & Mullen, 2004; Mahroof et al., 2010). Stored product insects also disperse among habitat patches within a food facility (Campbell et al., 2002). However, up to this point, it has been largely unknown whether stored product insects are also bringing microbes with them as they disperse, which is important because of the food safety implications. For the first time, we have documented that *L. serricorne* and *S. oryzae* will readily vector bacteria and fungi when introduced into new environments after even extended dispersal periods in sterilized transit environments. We generally found increasing dispersal periods resulted in fewer and less diverse microbes being vectored by both species, though a significant amount was still vectored by each and this pattern was more pronounced for *L. serricorne* than *S. oryzae*. Because this was done in sterilized environments where insects could not encounter or pick up new microbes, our results are likely conservative estimates of the potential vectoring capabilities of these stored product insects and the risk they could pose to food security. In addition, as time in patch increased, so too did microbial growth by multiple‐fold, likely as the combined result of movement by insects and spores around a dish and incubation at favorable temperatures. In prior work, *Aspergillus* spores have been detected on the cuticle of *L. serricorne* using scanning electron microscopy (Nakagawa et al., 2008), and from the current study, we know now *L. serricorne* as well as *S. oryzae* are capable of depositing viable spores from *Aspergillus* and other fungi into new food patches.

In the vectoring assay, we found individual trails of microbes created by the tarsal prints of *S. orzyae* and *L. serricorne*. This is likely from the physical deposition of spores while walking on the agar during the time in these factitious patches. Although we did not make direct observations, it is possible that other behaviors such as self‐grooming, antennation, resting, or frass deposition could be additional sources of microbes during the time that insects are on the agar (Meurant, 2012), as spores may become dislodged from the insect or embedded in the agar during these behaviors. While deposition of spores through walking may be a passive way that microbes are being vectored, some insects have specialized structures for storing microbial symbionts (Dowd, 1992; Martinson, 2020), and it could be that the spore density on the cuticle varies by the structural exoskeleton features for *S. oryzae* and *L. serricorne*. In other systems, the microbial abundance of wild‐caught butterflies depended on the body part, age, sex, and species, with microbial abundance greater on the tarsi and proboscis than on the thorax (Olson et al., 2023). Therefore, in the future, we should evaluate where spore density is highest anatomically on these species.

Indeed, our study shows that *L. serricorne* is more affected by dispersal period after leaving a food patch than *S. oryzae* in its ability to vector microbes. This is despite the fact that anecdotally, we consistently observed *S. oryzae* alive at 3 days whereas there was some occasional mortality by 5 days. The microbial growth on media containing *L. serricorne* decreased as dispersal period increased while it remained relatively more constant on media colonized by *S. oryzae*. These results suggest that *L. serricorne* is more sensitive to the stochastic effects experienced during a dispersal period and may be less effective at vectoring microbes to new food patches compared to *S. oryzae*. This could be due to differences in their dispersal behavior, cuticular composition and anatomy, or their mobility. Although less microbial growth and lower morphotype richness were observed with *L. serricorne*, their ability to disperse over long distances may enable them to vector microbes between distant food patches and even between different agroecosystems. For instance, evidence has shown that *L. serricorne* is a strong flyer and highly mobile, dispersing from food facilities to other habitats in the field (Shinoda & Fujisaki, 2001) and is thus a landscape‐level threat (Arthur et al., 2014; Edde, 2019). Campbell et al. (2002) studied an index of dispersal for pheromone trap captures in a warehouse for *L. serricorne* and found large numbers of insects in areas away from packaged food and food products. Collectively, the results of the current study suggest that *L. serricorne* dispersal likely brings a community of microbes, which suggests microbes and stored product insects should be managed in concert to ameliorate the risks from each.

In addition, we found that morphotype richness vectored by *L. serricorne* and *S. oryzae* varies with dispersal period as well. For example, the morphotype richness of microbes vectored by both *L. serricorne* and *S. oryzae* decreased with dispersal period, though in this case, the pattern was more pronounced for the former. Additionally, time in patch significantly affected morphotype richness of microbes vectored by both species, with greater diversity at 5 days compared to 3 days and more so for *L. serricorne* than *S. oryzae* at least at the 0 h dispersal but not subsequent dispersal periods. This suggests that there may be complex interactions among dispersal period, time in patch, microbial morphotype richness, and mobility. In particular, because *S. oryzae* is highly mobile through walking (Morrison et al., 2021), it is possible there are fewer gains in species richness from 3 to 5 days, while during this time *L. serricorne* is able to increase its species richness because of its lower baseline walking mobility (Ponce, 2023; Ponce, Sierra, et al., 2023). An alternative explanation is that by having a lower baseline mobility, there are fewer chances of microbes becoming dislodged, thus there are greater stochastic effects which do end up becoming dislodged from *L. serricorne* as more time passes. It is likely that further research is needed to understand these interactions more fully. It is also possible the conditions in the chamber and on the agar are selecting for certain microbial species, and the limited space and media hinders or abrogates growth of microbes on the dish. For example, at 3 days, the microbial population was less diverse due to the limited time for growth and interaction, and plates were never filled. By contrast, at 5 days, the microbial population exhibited greater diversity but plates were often (though not always) fully covered, suggesting possible limitation. In some cases, there were dominant microbial species such as *Aspergillus* spp., which appeared to be suppressing the growth of other microbial species, leading to reduced microbial diversity.

A variety of microbes can be found in grain, including bacteria, fungi, and yeasts. Some of the most common bacteria include *Bacillus*, *Enterobacter*, and *Pseudomonas*, while some of the most common fungi include *Aspergillus*, *Fusarium*, and *Penicillium* spp. (Magan et al., 2003). The presence of these microbes generally has a negative effect on grain quality and safety. For instance, some bacteria and fungi can produce toxins that are harmful to human and animal health and can also reduce the quality and shelf life of the grain. In addition, microbial growth can cause elevated temperature and moisture content that are ideal for insect development, termed hotspots (Ponce et al., 2021). But, there are also beneficial fungi that can be used antagonistically to occupy the same niche as mycotoxin‐producing fungi, but without its deleterious effects (Wambacq et al., 2016). In fact, in this study, we found that *L. serricorne* and *S. oryzae* vector *Aspergillus*, *Fusarium*, and *Penicillium* spp. One of the most frequent microbial species found in this study was *Aspergillus flavus* (Eurotiales: Trichocomaceae), which is partly responsible for the production of a mycotoxin called aflatoxin B1 and secondary metabolites that help the fungus colonize and infect maize kernels (Antiga et al., 2020). Our results demonstrate that stored product insects such as *L. serricorne* and *S. oryzae* may be contributing to the spread of *A. flavus* as they disperse. This could be particularly relevant to the prevalence of *A. flavus* as the fungus may benefit from a vector to spread and infect new hosts. This is perhaps also a key potential indicator finding for other microbial species that may share a similar disease cycle. Other work has found that *Tyrophagus putrescentiae* (Acaridae) infestations influenced the appearance of new fungal species and significantly induced higher mycotoxin concentrations (Vogel et al., 2021). Fungal cues from *A. flavus* affect the foraging ecology of both primary and secondary stored product insects (Ponce et al., 2022; Ponce, 2023; Ponce, Sierra, et al., 2023), and progeny production by *S. zeamais* increased in the presence of *Fusarium verticillioides* (Usseglio et al., 2020). *Lasioderma serricorne* is associated with a long‐standing symbiosis with the yeast‐like fungus, *Symbiotaphrina kochii* (Martinson, 2020). Future work should address whether the strains of *A. flavus* being spread by *L. serricorne* and *S. oryzae* are capable of producing mycotoxins.

Even though this study was done under controlled environmental conditions, temperature and humidity are important factors that can affect insect–microbe interactions in the postharvest supply chain. Beti et al. (1995) found that the combination of *S. zeamais* with maize positively increased grain moisture, and increased grain moisture was correlated with higher aflatoxin content from *A. flavus*. In addition, some studies have shown that high temperatures can be used to manage the risk caused by insects by reducing the survival and reproduction of insect pests and thus reducing the risk of microbial contamination (Silva & Elliot, 2016; Skendžić et al., 2021). Similarly, low humidity can also reduce the survival and reproduction of some insect pests (Norhisham et al., 2013). Furthermore, pH levels, modified atmospheres (e.g., oxygen levels), and nutrients available are other important factors that could be affecting microbial growth as well as diversity. However, it is noteworthy that the optimal temperature and humidity conditions for insect pests and microbes may vary depending on the species involved and other factors, such as the type of commodity and the duration of storage. For example, grain commodities such as maize, sorghum, or wheat can have different physiological properties that may benefit the reproduction and diversification of different microbial species (Dabija et al., 2021; Singh et al., 2023).

In this study, we also evaluated the emigration of *S. oryzae* from microbe‐rich environments to understand how this may change the dynamics of microbial growth and species richness over different dispersal times and time foraging in food patch. We found that there was reduced microbial growth with increasing dispersal time. In addition, we also observed quick saturation of the plate with microbes already by 3 days after introduction and little change after 5 days. This suggests that even over the extended dispersal periods, spores will readily dislodge in the new environment and fungi will colonize quickly if conditions are favorable. Prior work found maize exposure by *S. zeamais* from 7 up to 21 days cumulatively with weevils previously inoculated with *A. flavus* increased the aflatoxin B1 content of the commodity (Beti et al., 1995). Those authors concluded that *S. zeamais* facilitated the growth of *A. flavus* in maize over time by increasing the surface area susceptible to fungal infection and increasing the moisture content from insect activity.

In addition, future work should evaluate the effect of habitat foraging preferences during dispersal and how that affects choice and subsequent progeny production by *L. serricorne*. Overall, our study sheds some light on the relative risk of microbial contamination by two stored product insect species (e.g., an internal‐infesting and external‐infesting pest) in postharvest commodities. Our findings suggest that both species (*L. serricorne* and *S. oryzae*) pose food safety risks, but that further information is required to determine why different microbial communities were associated with each insect and how the different microbial communities and the different life histories of each insect contribute to risk. Nonetheless, overall this information is useful for food safety and pest management in the postharvest supply chain, as it could help identify which insect species pose the greatest risk of microbial contamination and which ones should be targeted for management measures. Ultimately, this research helps highlight an underestimated risk to the food safety of the postharvest supply chain by the joint action of insects and microbes.

## AUTHOR CONTRIBUTIONS

**Marco A. Ponce:** Conceptualization (equal); data curation (lead); formal analysis (equal); funding acquisition (equal); investigation (lead); methodology (lead); project administration (equal); resources (equal); validation (equal); visualization (equal); writing – original draft (lead); writing – review and editing (equal). **Jacqueline M. Maille:** Data curation (supporting); formal analysis (supporting); investigation (supporting); methodology (equal); validation (supporting); writing – review and editing (equal). **Ian Stoll:** Investigation (supporting); methodology (supporting); writing – review and editing (equal). **Avery James:** Investigation (supporting); methodology (supporting); writing – review and editing (supporting). **Alexander Bruce:** Investigation (supporting); methodology (supporting); project administration (supporting); validation (supporting); visualization (supporting); writing – review and editing (equal). **Tania N. Kim:** Methodology (supporting); project administration (supporting); supervision (equal); validation (equal); writing – review and editing (equal). **Erin D. Scully:** Funding acquisition (supporting); methodology (supporting); project administration (supporting); writing – review and editing (equal). **William R. Morrison:** Conceptualization (equal); data curation (supporting); formal analysis (equal); funding acquisition (equal); investigation (equal); methodology (equal); project administration (lead); resources (equal); supervision (lead); validation (supporting); visualization (supporting); writing – review and editing (equal).

## CONFLICT OF INTEREST STATEMENT

The authors declare that the research was conducted in the absence of any commercial or financial relationships that could be construed as a potential conflict of interest.

## Supporting information


Appendix S1

## Data Availability

Ag Data Commons. https://doi.org/10.15482/USDA.ADC/1529590. Accessed 2023‐09‐23 (Ponce, Maille, et al., 2023).
