# Supplementary material for: Microbial vectoring capacity by internal‐ and external‐infesting stored product insects after varying dispersal periods between novel food patches: An underestimated risk
Source: Ecol Evol. 2024 Jun 25;14(6):e11368. doi: 10.1002/ece3.11368 (PMC11199339; doi:10.1002/ece3.11368)
Supplement: Supplementary file 1 — Appendix S1 [file ECE3-14-e11368-s001.docx]

**Appendices**


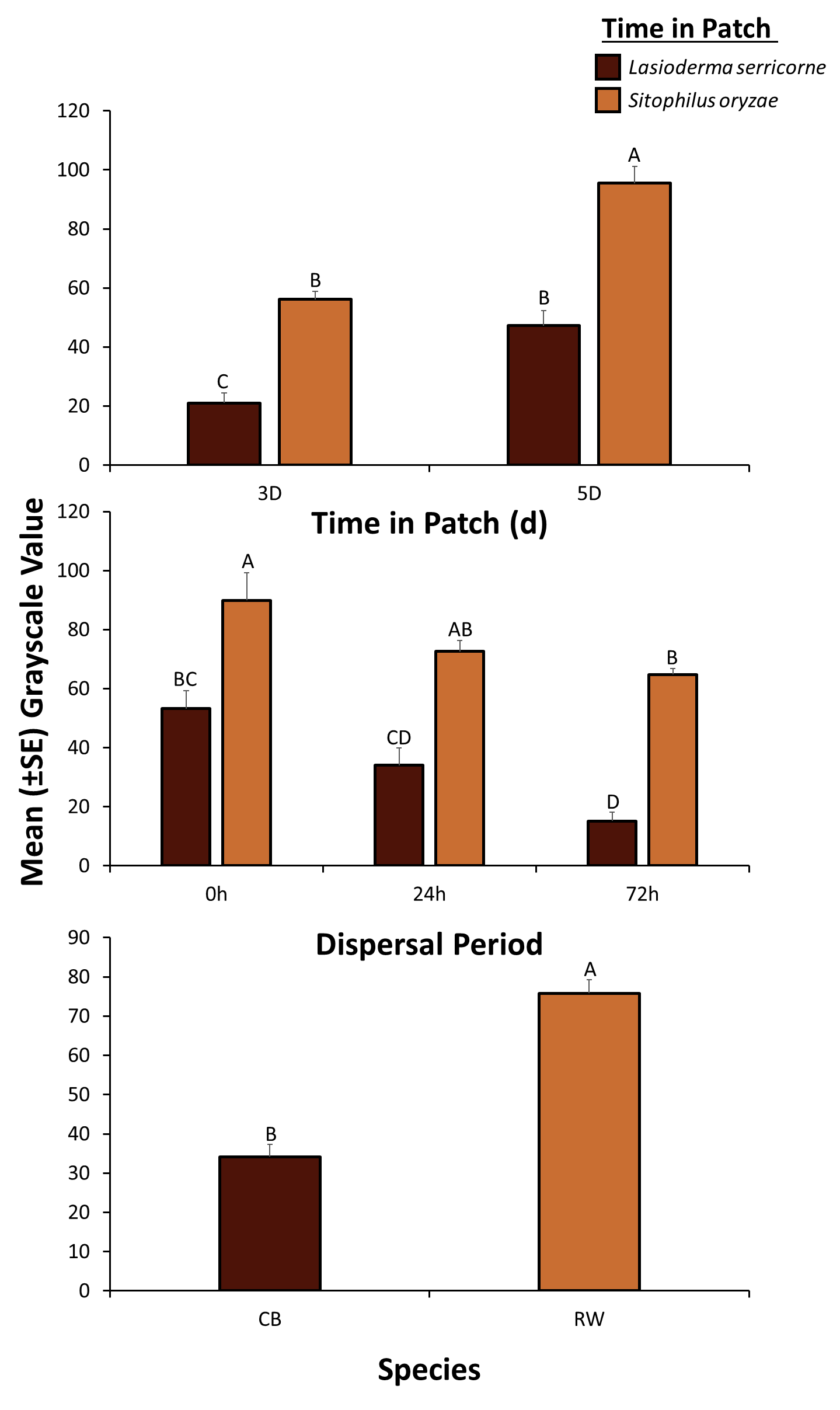


**Figure S1.** Direct comparisons of mean microbial growth of vectored microbes between *L. serricorne* (CB, red) and *S. oryzae* (RW, orange), depending on time in patch (top panel; 3 d or 5 d), dispersal period (middle panel; 0, 24, or 72 h), or overall by species after introduction in factitious foraging patches consisting of potato dextrose agar dishes that were photographed using a 3D-imaging system and processed with ImageJ. There were a total of n = 30 replicate adults tested per bar. Bars with shared letters are not significantly different from each other (Tukey HSD, α = 0.05). The grayscale value can range from 0, indicating no microbial growth, to 255, indicating the full agar dish was covered with microbes.


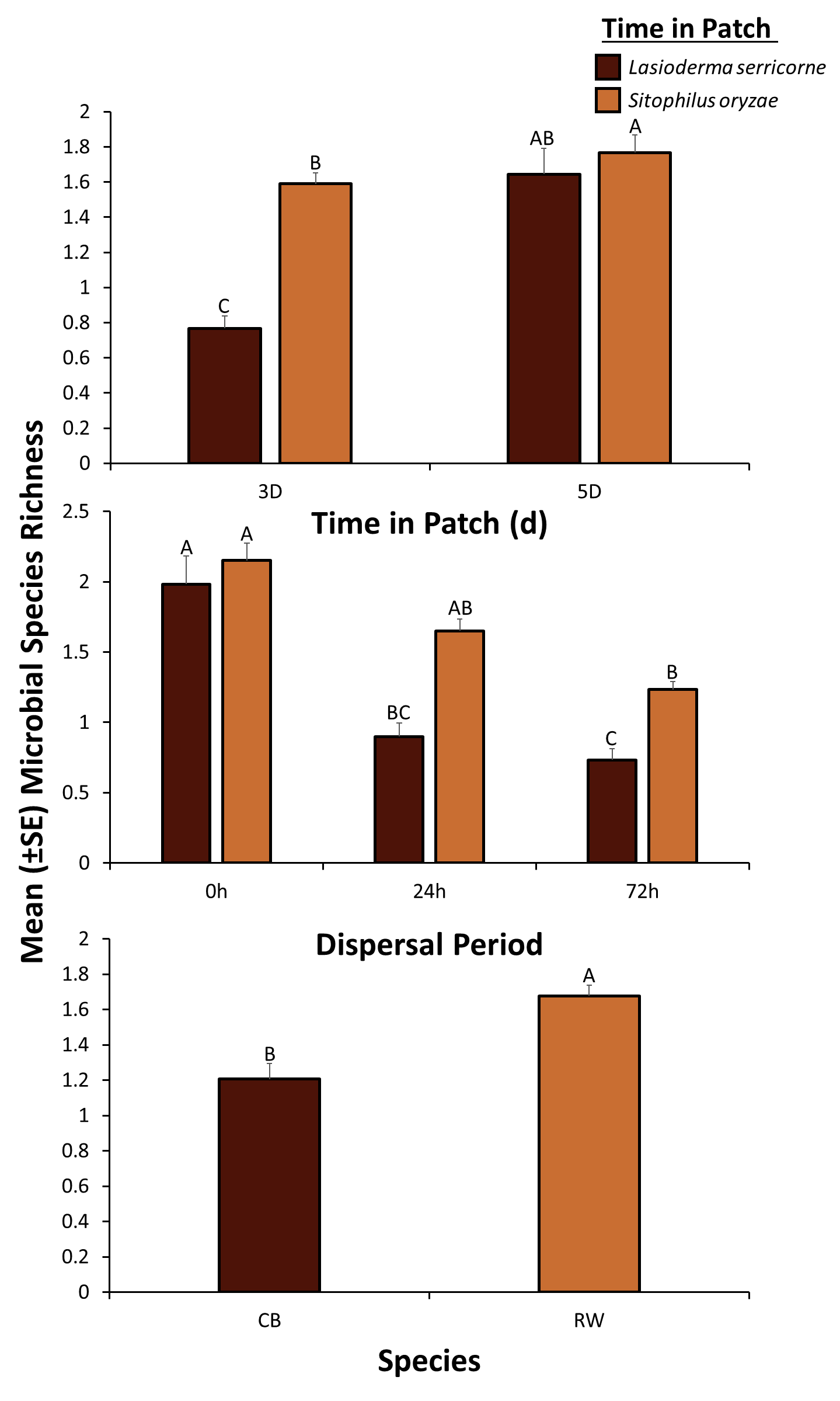


**Figure S2.** Direct comparisons of mean species richness of vectored microbes between *L. serricorne* (CB, red) and *S. oryzae* (RW, orange), depending on time in patch (top panel; 3 d or 5 d), dispersal period (middle panel; 0, 24, or 72 h), or overall by species after introduction in factitious foraging patches consisting of potato dextrose agar dishes that were photographed using a 3D-imaging system and processed with ImageJ. There were a total of n = 30 replicate adults tested per bar. Bars with shared letters are not significantly different from each other (Tukey HSD, α = 0.05). The grayscale value can range from 0, indicating no microbial growth, to 255, indicating the full agar dish was covered with microbes.

| **Table S1.** Preliminary summary of ANOVA models for mean grayscale value (microbial growth) and microbial diversity (e.g., species richness) after the introduction of *L. serricorne* and *S. oryzae* from colony population on PDA agar dishes after a 0, 24, or 72 h dispersal period. | | | | | | |
| --- | --- | --- | --- | --- | --- | --- |
|  | **Mean Grayscale Value** | | |  | **Species Richness** | |
| Variable | df | *F* | *P* |  | *F* | *P* |
|  |  | | | | | |
| Dispersal Period | 2 | 1.32 | 0.27 |  | 16.3 | 0.0001 |
| Time in Patch | 1 | 3.20 | 0.08 |  | 18.6 | 0.0001 |
| Species | 1 | 6.4 | 0.01 |  | 4.80 | 0.02 |
| Dispersal Period: Time in Patch | 2 | 0.25 | 0.78 |  | 9.31 | 0.001 |
| Dispersal Period: Species | 2 | 1.2 | 0.32 |  | 7.05 | 0.001 |
| Time in Patch: Species | 1 | 19.81 | 0.0001 |  | 3.17 | 0.08 |
| 3-way interaction | 2 | 5.61 | 0.0001 |  | 17.87 | 0.0001 |
| Residuals | 258 |  |  |  |  |  |
